# Prevalence and species distribution of the low-complexity, amyloid-like, reversible, kinked segment structural motif in amyloid-like fibrils

**DOI:** 10.1016/j.jbc.2021.101194

**Published:** 2021-09-16

**Authors:** Michael P. Hughes, Lukasz Goldschmidt, David S. Eisenberg

**Affiliations:** 1 Department of Cell and Molecular Biology, St Jude Children's Research Hospital, Memphis, Tennessee, USA; 2 Department of Biochemistry, University of Washington, Seattle, Washington, USA; 3 Department of Biological Chemistry, UCLA-DOE Institute, HHMI, and Molecular Biology Institute, UCLA, Los Angeles, California, USA

**Keywords:** LARKS, amyloid, phase-separation, membraneless organelles, low-complexity domains, GO, Gene Ontology, IDR, intrinsically disordered region, LARKS, low-complexity amyloid-like reversible kinked segment, LCD, low-complexity domain, LARKS ∩ LCD protein, protein that has at least one LCD and for which the fraction of LARKS is greater than average for its proteome, LARKS ∩ LCD residue, an amino acid residue that is a predicted LARKS within a LCD, MLO, membraneless organelle, PDB, Protein Data Bank, SG, stress granule

## Abstract

Membraneless organelles (MLOs) are vital and dynamic reaction centers in cells that compartmentalize the cytoplasm in the absence of a membrane. Multivalent interactions between protein low-complexity domains contribute to MLO organization. Previously, we used computational methods to identify structural motifs termed low-complexity amyloid-like reversible kinked segments (LARKS) that promote phase transition to form hydrogels and that are common in human proteins that participate in MLOs. Here, we searched for LARKS in the proteomes of six model organisms: *Homo sapiens*, *Drosophila melanogaster*, *Plasmodium falciparum*, *Saccharomyces cerevisiae*, *Mycobacterium tuberculosis*, and *Escherichia coli* to gain an understanding of the distribution of LARKS in the proteomes of various species. We found that LARKS are abundant in *M. tuberculosis*, *D. melanogaster*, and *H. sapiens* but not in *S. cerevisiae* or *P. falciparum*. LARKS have high glycine content, which enables kinks to form as exemplified by the known LARKS-rich amyloidogenic structures of TDP43, FUS, and hnRNPA2, three proteins that are known to participate in MLOs. These results support the idea of LARKS as an evolved structural motif. Based on these results, we also established the LARKSdb Web server, which permits users to search for LARKS in their protein sequences of interest.

A new area of cell biology is the study of membraneless organelles (MLOs) in the organization of cellular structures and metabolism. Many MLOs are RNA and protein assemblies that fulfill specific functions for the cell. Examples of MLOs in human cells include P bodies that degrade mRNA, stress granules (SGs) that store mRNA during stresses, and the nucleolus that processes rRNA. MLOs also function in other organisms such as germline P granules in *Caenorhabditis elegans* and SGs in yeast. The aforementioned organelles are not enveloped by membranes to partition them from the cytoplasm but instead organize through multivalent networks of homotypic and heterotypic reversible interactions between proteins and nucleic acids (1, 2). These reversible networks allow MLOs to be dynamic. They may assemble and disassemble in response to stimuli like SGs do in response to stresses and dissolve as stresses subside. Proteins in MLOs often contain low-complexity domains (LCDs) that help to drive reversible organization (3, 4).

LCDs are regions of proteins with significant biases for one or a few amino acids. An example is the LCD of FUS where four amino acids glycine, tyrosine, serine, and glutamine account for 80% of the LCD composition. The LCD from FUS has also been termed an intrinsically disordered region (IDR) or a prion-like domain. The LCD of FUS is an IDR because for most of the time it lacks a defined globular structure. In fact, LCDs are a reasonable proxy for IDRs (5), but this is not always the case (*e.g.*, collagen proteins that are low in sequence complexity but have a defined structure). Prion-like domains technically refer to a domain that resembles a yeast prion sequence that is rich in asparagine and glutamine (6). Hence, yeast prions are low in complexity by definition, and prion-like domains are typically disordered until forming amyloid fibrils. FUS was identified as a prion-like domain in humans by a search for proteins with sequence biases similar to yeast prions, alongside a number of other proteins that are involved in SGs including hnRNPA1, hnRNPA2, and TDP43 (4, 6, 7, 8). However, not all LCDs are prion-like domains; arginine–glycine-rich LCDs are common in RNA-binding proteins and less prone to aggregate than other LCDs (9).

Remarkably, when the LCDs from hnRNPA1, hnRNPA2, FUS, and TDP43 are purified, they undergo liquid–liquid phase transition and eventually form hydrogels composed of amyloid-like fibrils (4, 10, 11, 12). Phase transitions are also the governing force forming MLOs, and some proteins contribute to MLO organization through their IDRs and LCDs (13). The first phase transition is a liquid–liquid phase separation that leads to a protein-rich phase compared with the surrounding bulk solvent. Some proteins may undergo a second phase transition from liquid to solid to form amyloid fibrils (10, 12, 14, 15). hnRNPA1, hnRNPA2, FUS, and TDP43 have all been found aggregated in amyloid diseases, and MLOs have been proposed as a crucible for driving fibril formation in amyotrophic lateral sclerosis (16, 17, 18).

Amyloid fibrils have long been associated with disease and neurodegeneration, but there are now abundant examples of functional amyloid. Examples include curli fibrils made by *Escherichia coli* to construct biofilms, prions in yeast to alter phenotypes, and premelanosome protein granules in humans to make pigment (19, 20). In all these examples, the organism takes advantage of the ability of proteins to form fibrils based on mated β-sheets. The fibrils formed from the LCDs of FUS, hnRNPA1, and hnRNPA2 are notable because these amyloid-like fibrils are labile (4, 11, 12) and easily reversed by elevated temperature, changes of solvent conditions, or dilution. This lability enables amyloid fibrils formed by LCDs to participate in the organization and dynamics of MLOs but contrast with the detrimental stability of pathogenic amyloid.

In previous work, we identified short adhesive motifs termed low-complexity amyloid-like reversible kinked segments (LARKS) that capture the reversible fibril behavior of these proteins (15). LARKS allow proteins to form β-sheet–rich structures that hydrogen bond along the fibril axis to create amyloid-like fibrils, but the LARKS introduce sharp kinks in the peptide backbone, which interrupts the pleated β-sheets (see Discussion section). This structure contrasts with the adhesive elements we find in amyloid fibrils called steric zippers that form extended and pleated β-sheets with interdigitated side chains giving amyloid fibrils their typical stability (21). The LARKS structure is stable enough to form a fibril but avoids the irreversibility of a steric zipper and appears to function in the organization of MLOs.

We computationally searched for LARKS motifs in the human proteome by threading protein sequences onto known LARKS structures and using a Rosetta energy algorithm to predict if each segment can adopt a LARKS structure (15, 22). This search through the human proteome found that LARKS are common in LCDs of proteins found in MLOs. Here, we extend this search for LARKS in the organisms *E. coli*, *Mycobacterium tuberculosis*, *Saccharomyces cerevisiae*, *Plasmodium falciparum*, and *Drosophila melanogaster* to compare with the distribution of LARKS in the *H. sapiens* proteome. These model organisms were chosen because they are well studied and cover an array of complexity. *E. coli* and *M. tuberculosis* are both prokaryotes, but *M. tuberculosis* is an intracellular parasite. *P. falciparum* was chosen as a eukaryotic parasite, *S. cerevisiae* as a single-celled eukaryote, and *D. melanogaster* as an example of multicellular eukaryote. We compare the LARKS predictions for these model organisms to make several findings.

Threading reveals that not all species have LARKS-rich LCD domains, as the LCDs of *S. cerevisiae* and *P. falciparum* are not enriched in LARKS. This correlates with a lack of glycine in their LCD amino acid bias. We go on to provide examples of how LARKS and amino acid composition influence amyloid structure. Overall, this work helps us to understand the roles that LARKS play in biology. We make all our LARKS predictions publicly available with our online database LARKSdb (http://servicesn.mbi.ucla.edu/LARKSdb/).

## Results

### LARKS-rich proteins overlap with LCD-containing proteins in most species

**Figure 1:**
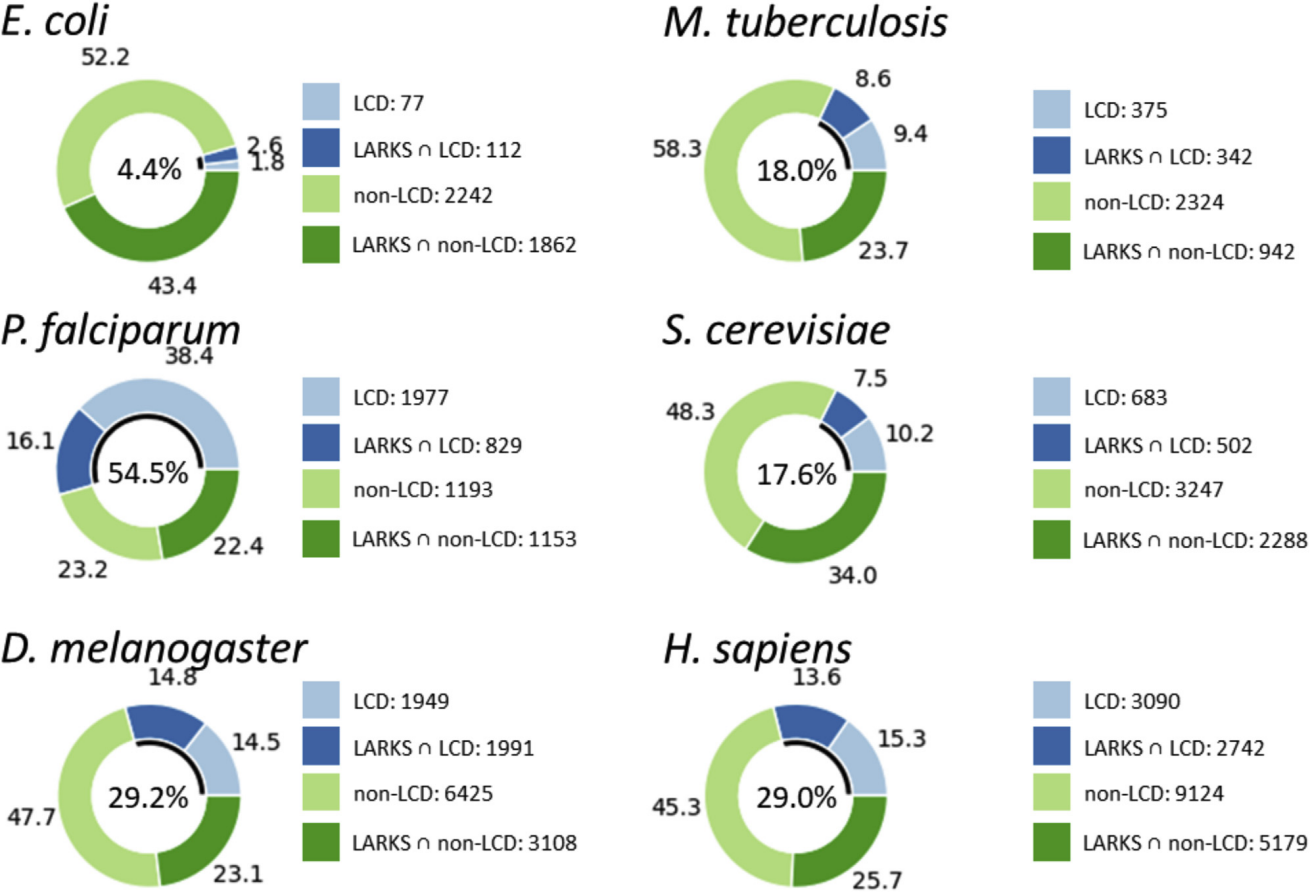
**LARKS and LCDs of proteins in six analyzed proteomes.** The center of each donut chart gives the percentage of proteins in the proteome that have an LCD and is represented by the *black arc* in the inside of the donut chart. The *outer ring* of the donut chart shows the percentage of proteins that are LARKS rich with an LCD (*dark blue*: LARKS ∩ LCD), just LCD-containing (*light blue*: LCD), LARKS rich without having an LCD (*dark green*: LARKS ∩ non-LCD), or non-LCD–containing (*light green*: non-LCD). The integers listed next to the keys give precise values for the number of proteins in each category for their respective proteomes. LARKS, low-complexity amyloid-like reversible kinked segment; LCD, low-complexity domain.

Past work found that the proteins with the most LARKS overlap with LCD-containing proteins in humans (15). We hypothesized that LARKS are an enriched motif across LCDs of organisms that use them to organize MLOs, as in *H. sapiens*, and searched our threading data to find LARKS-rich proteins. We defined a LARKS-rich protein as any protein having more LARKS per 100 residues than the average value for the proteome of that species. We found that most, but not all, LCD-containing proteomes are rich in LARKS. In *H. sapiens*, we see that 47% of the LCD-containing proteins are LARKS rich, whereas only 36% of proteins without LCDs were considered LARKS rich (Fig. 1). This indicates enrichment, and *p* values from bootstrapping confirmed that LCD-containing proteins in *H. sapiens* are significantly more likely to be LARKS rich than if the proteins did not have an LCD: *p* value = 1.0 × 10^−4^ (Fig. S1). Next, we compared LARKS enrichment in LCD proteins from *H. sapiens* to other proteomes. *E. coli* was chosen as a model bacterial organism with a minimal proteome that has very few LCDs. In fact, only 4.4% of *E. coli* proteins have an LCD, but even that small sample is still significantly enriched in LARKS (*p* = 3.0 × 10^−4^) (Fig. 1 and Fig. S1). To compare to another higher multicellular organism, we chose *D. melanogaster* and find it has similar LCD and LARKS content to *H. sapiens*. *D. melanogaster* LCD-containing proteins are also enriched in LARKS (*p* = 3.0 × 10^−4^). Next, we compared the *S. cerevisiae* proteome, which is a single-celled eukaryote known for its extensive LCDs that form prions. About 17.6% of the *S. cerevisiae* proteome has LCDs, and to our surprise, they are not significantly enriched in LARKS (*p* = 0.28). LARKS are a structural motif abundant in LCD-containing proteins of *H. sapiens* and *D. melanogaster* but not in *S. cerevisiae*.

Following this, we compared other single-celled organisms with substantial LCD content and chose the two intracellular obligate parasites: The *M. tuberculosis* bacterium responsible for tuberculosis and the *P. falciparum* eukaryote responsible for malaria. *M. tuberculosis* has a proteome where 18% of proteins have an LCD, and LARKS-rich proteins are significantly enriched in the LCD-containing proteins (*p* = 1.0 × 10^−4^). The opposite was found with *P. falciparum* where 54.5% of proteins contain LCDs and of those LARKS-rich proteins are significantly underrepresented (*p* = 1.0 × 10^−4^). Contrary to the LARKS ∩ LCD-rich proteomes of *E. coli*, *M. tuberculosis*, *D. melanogaster*, and *H. sapiens*, we consider *S. cerevisiae* and *P. falciparum* to have LARKS ∩ LCD-poor proteomes. In summary, the analysis shows that proteomes differ markedly in their proportion of LARKS ∩ LCD proteins.

### LARKS residues overlap with LCDs in LARKS-rich proteomes

The aforementioned analysis found the extent to which LARKS motifs are included in whole proteins that contain an LCD. Next, we examine the extent to which the LCDs of proteomes are enriched in LARKS. We also examine the extent to which proteins contain LCDs where LARKS do not reside in the LCD. To do so, we summed the residues of each proteome and counted the number of residues that are in both an LCD and a LARKS (Table 1) and called these LARKS ∩ LCD residues. The number of LARKS ∩ LCD residues is divided by the total number of residues to find the fraction of LARKS ∩ LCD residues in a proteome. We use bootstrapping methods to give a 95% confidence interval based on the variance of the data for the actual fraction of LARKS ∩ LCD residues (see Experimental procedures section). We compare this number to the expected probability of this intersection calculated by multiplying the fraction of LCD residues by the fraction of LARKS residues. If the expected intersection of LARKS ∩ LCD residues is less than the 95% confidence interval for the actual intersection, we consider the LCDs to be enriched in LARKS.

**Table 1:** Residue content of proteomes

Species	Number of proteins	Number of LCD proteins	% LARKS	% LCD	% Glob
*E. coli*	4293	199	2.4	0.6	86.9
*M. tuberculosis*	3983	717	3.9	5.4	76.4
*P. falciparum*	5152	2806	0.8	11.3	78.3
*S. cerevisiae*	6720	1185	1.7	2.5	78.2
*D. melanogaster*	13,473	3940	2.3	5.7	72.5
*H. sapiens*	20,135	5832	2.1	3.9	71.2

Species	% LARKS ∩ LCD		% LARKS ∩ Glob		% LCD ∩ Glob	
	Actual	Expected	Actual	Expected	Actual	Expected
*E. coli*	0.000 ≤ 0.022 ≤ 0.050	0.015	1.843 ≤ 1.853 ≤ 1.866	2.072	0.431 ≤ 0.437 ≤ 0.445	0.537
*M. tuberculosis*	1.236 ≤ 1.267 ≤ 1.296	0.214	1.910 ≤ 1.935 ≤ 1.955	3.009	1.693 ≤ 1.730 ≤ 1.763	4.149
*P. falciparum*	0.080 ≤ 0.088 ≤ 0.096	0.092	0.486 ≤ 0.490 ≤ 0.495	0.64	4.641 ≤ 4.661 ≤ 4.683	8.833
*S. cerevisiae*	0.048 ≤ 0.062 ≤ 0.073	0.041	1.054 ≤ 1.063 ≤ 1.072	1.298	0.969 ≤ 0.981 ≤ 0.992	1.925
*D. melanogaster*	0.346 ≤ 0.356 ≤ 0.365	0.128	1.077 ≤ 1.082 ≤ 1.092	1.639	2.016 ≤ 2.03 ≤ 2.043	4.123
*H. sapiens*	0.180 ≤ 0.186 ≤ 0.194	0.081	1.124 ≤ 1.129 ≤ 1.133	1.491	1.223 ≤ 1.232 ≤ 1.240	2.748

The percent of residues in each proteome that were found to be in LARKS, LCDs, or globular regions is shown in columns 4 to 6 of the top table. The table below gives the statistics of overlapping residue types to compare the actual to the expected. Shown in the actual column are the fractions of residues meeting the criteria of the form of a 95% confidence interval with the following percentiles 2.5% ≤ actual overlap ≤ 97.5%. Confidence intervals of the actual value reflect the variance of the data from 100 rounds of bootstrapping (see Experimental procedures for details). The expected intersection value is the fraction of LARKS residues multiplied by the fraction of LCD residues. If the expected value is below the given 2.5% confidence interval for the actual value, then LARKS are enriched in LCDs. If the expected value is above the 97.5% confidence interval, then LARKS are depleted in LCDs compared with what would be expected for that organism. The same methodology is repeated to find LARKS ∩ Glob and LCD ∩ Glob. Comparing the actual LARKS ∩ LCD with the expected indicates that LARKS are enriched in LCDs in all organisms studied except *P. falciparum*.

Overall, these results show that for *E. coli*, *M. tuberculosis*, *D. melanogaster*, and *H. sapiens*, the actual number of residues that intersect between LARKS and LCDs is much greater than expected by probability (Table 1). This agrees with the previous analysis that LARKS are enriched in LCDs and that the LARKS reside within the LCD residues in these organisms. *S. cerevisiae* shows a slight enrichment, and *P. falciparum* has no enrichment of LARKS in LCDs, as the expected value is within the given 95% confidence interval. This largely agrees with the data from Figure 1 and goes further by showing that LARKS reside in the actual LCDs of organisms that have LARKS ∩ LCD proteins.

We searched for LARKS in LCDs because LCDs are typically disordered, and LARKS need to be solvent exposed to allow interactions that affect function. While LCDs are a reasonable proxy for disorder, they are not always perfect (5). Therefore, we also repeated the same analysis but looked for predicted globular and disordered regions as defined by the algorithm Glob (23). We call LARKS in predicted globular regions LARKS ∩ Glob, and for each proteome, the actual number of LARKS ∩ Glob is lower than expected in predicted globular regions (Table 1). This indicates enrichment of the LARKS motifs in IDRs of proteins. In fact, even in proteomes that lack LARKS ∩ LCD proteins, we see that LARKS ∩ Glob residues are rarer than expected. We interpret this to mean LARKS are excluded from globular regions indicating the motif preference to be in IDRs regardless if that IDR is an LCD or not. To gain insight into why LCDs in some proteomes are enriched in LARKS, whereas others are not, we looked at LCD amino acid compositions of proteomes.

### Amino acid biases are different depending on species

**Figure 2:**
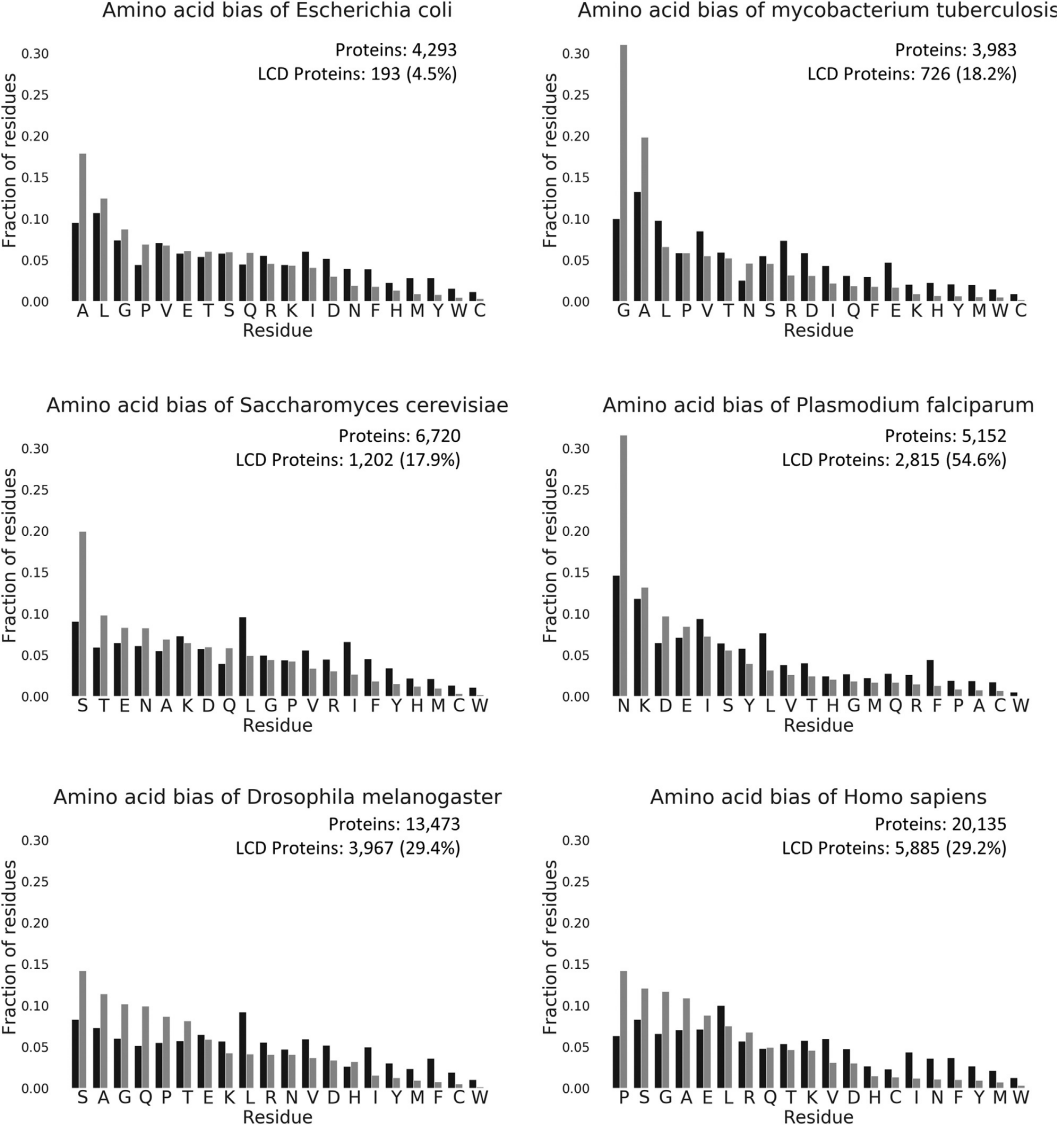
**Amino acid bias of proteomes.***Black bars* represent the fraction of each proteome made up of that amino acid. *Gray bars* represent the fraction of LCD residues within the proteome made up by each residue. Amino acids are ordered by the most common amino acids found in that proteome's LCDs. LCD, low-complexity domain.

To gain understanding of the variation among proteomes of the extent of LARKS in LCDs, we examined the amino acid composition of LCDs in different proteomes. We found glycine to be the most common residue in predicted LARKS. This abundance of glycine is consistent with LARKS structures where the allowed phi–psi angles form kinks in the LARKS while maintaining hydrogen bonds along the fibril axis (15). In the proteomes with LARKS-rich LCDs (*E. coli*, *M. tuberculosis*, *D. melanogaster*, and *H. sapiens*) (Fig. 1), we find that glycine is among the five most common residues (Fig. 2). Asparagine is not one of the five most common residues in these proteomes but is among the top five of the *S. cerevisiae* and *P. falciparum*. The presence of glycine in the top five residue amino acids of LARKS ∩ LCD-rich proteomes juxtaposed to its absence in LARKS ∩ LCD-poor proteomes is striking. To gain insight into the function of LARKS in LCDs, we studied amyloid structures from LARKS-rich and LARKS-poor proteins (see Discussion section).

## Discussion

LARKS are structural motifs that mediate labile interactions that form reversible amyloids and are associated with phase transitions. Here, we use more rigorous methodology to confirm our previous finding that LARKS are motifs enriched in LCDs and IDRs in *H. sapiens*. However, when we applied this same analysis to other organisms, we found a broad spectrum of LARKS content in the LCDs of various species. Across all proteomes analyzed, LARKS are depleted in globular regions (Table 1) indicating that the sharp kink inherent to LARKS is poorly accommodated in globular structure. However, LARKS are not enriched in the LCDs of all proteomes (Fig. 1 and Fig. S1), and we were curious if the presence of LARKS-rich proteins correlates with biology of the respective species' proteome as judged by a qualitative analysis of the Gene Ontology (GO) terms over represented in the LARKS-rich proteins of each proteome (Table S2).

**Figure 3:**
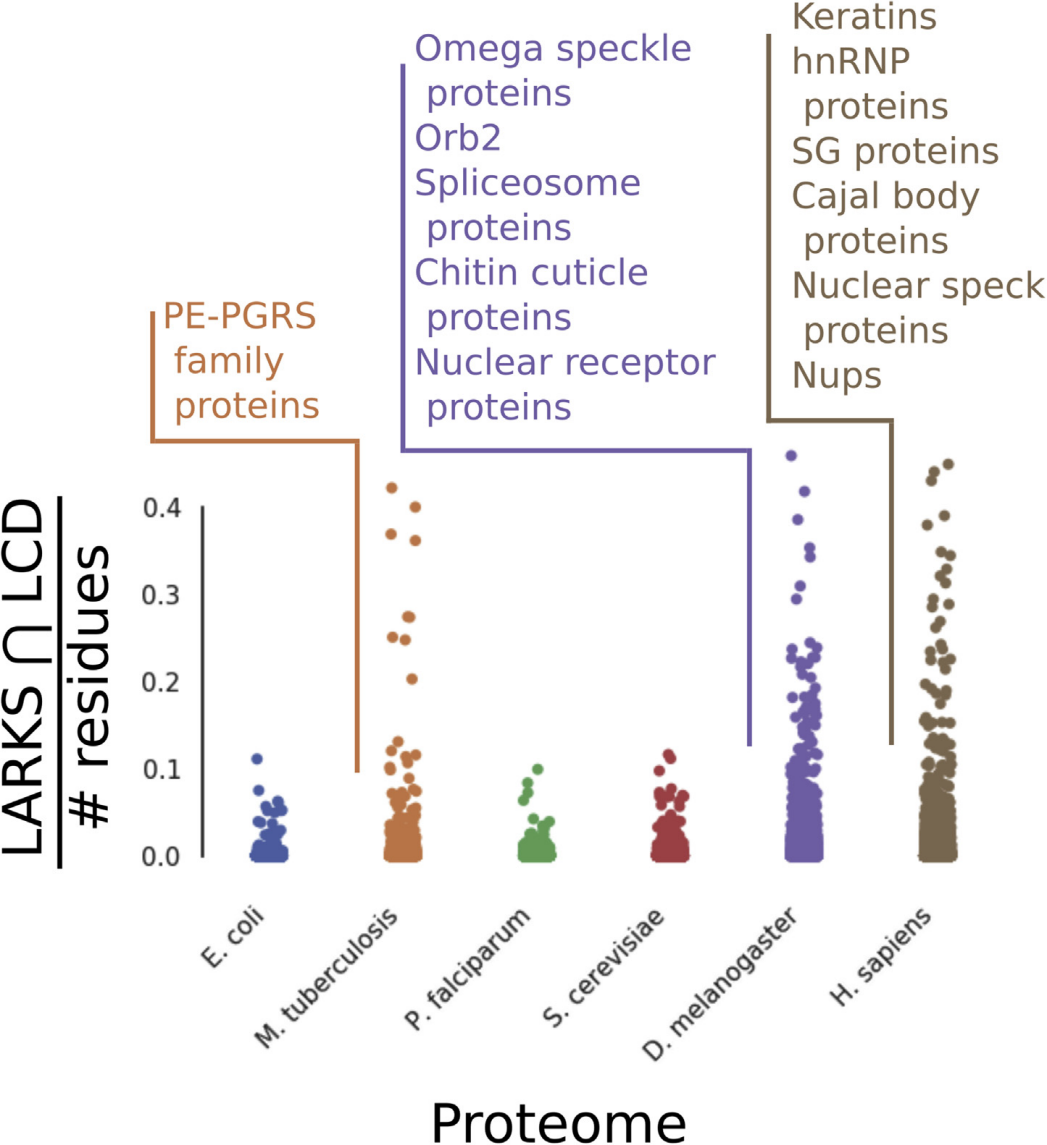
**Swarm plot of LARKS ∩ LCD proteins in the six analyzed proteomes.** Each *dot* represents a single protein and is placed on the *Y*-axis according to the number of LARKS ∩ LCD residues divided by the protein length. Examples of types of LARKS-rich proteins from *Mycobacterium tuberculosis*, *Drosophila melanogaster*, and *Homo sapiens* are listed and largely associated with MLOs. Examples are taken from Table S2. LARKS, low-complexity amyloid-like reversible kinked segment; LCD, low-complexity domain; MLO, membraneless organelle.

Of other proteomes we analyzed, *D. melanogaster* is most similar to *H. sapiens* in LARKS abundance and LCD content and closest to *H. sapiens* in evolutionary distance. GO terms are very similar for LARKS ∩ LCD proteins between the two organisms. Both proteomes are enriched for proteins involved in RNA binding, transcription factor binding, MLOs (*e.g.*, omega speckles, SGs, nuclear specks, and Cajal bodies), and extracellular matrix/cuticle formation (Fig. 3 and Table S2). In our 2018 analysis of LARKS in the human proteome, we identified that keratins are highly enriched in LARKS and posited that phase separation may be an important aspect of cuticle formation (15), and this was subsequently confirmed in later studies (24). GO for LARKS ∩ LCD proteins in *D. melanogaster* included a term for “structural constituent of the chitin-based cuticle,” which is the fly barrier equivalent. Chitin is a main component of the *D. melanogaster* exoskeleton and absent in *human skin*. It will be interesting to see if arthropods use LARKS akin to *H. sapiens* in cuticle formation.

**Figure 4:**
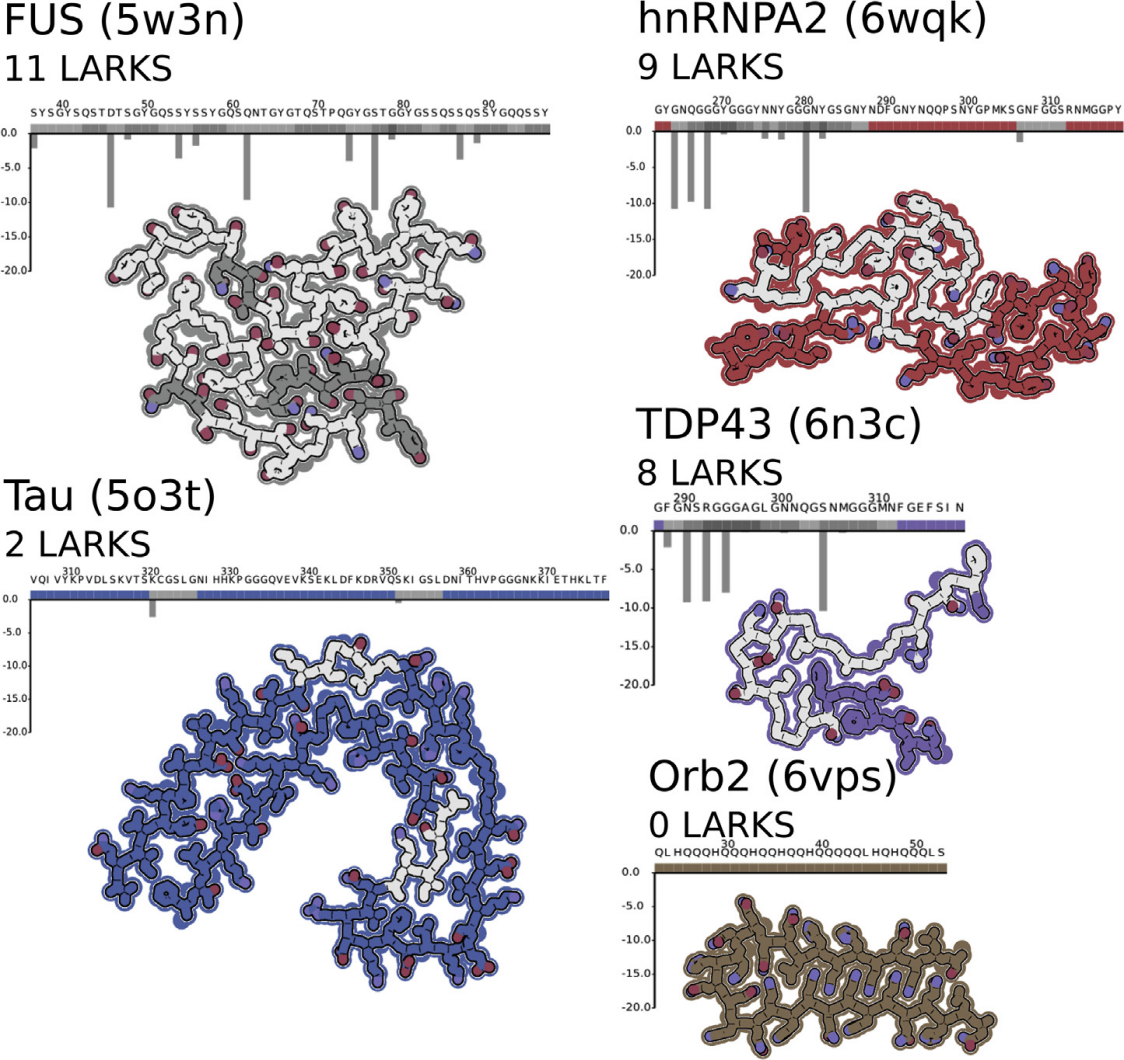
**LARKS alter amyloid structure.** All images are made from a single chain and a single layer of the fibril, being viewed down the fibril axis. LARKS predictions and sequences are shown above structures. *Gray boxes* above the sequence indicate predicted LARKS, and the corresponding residues in the protein images have a *gray* interior. There are three structures of LARKS-rich LCDs: from FUS, hnRNPA2, and TDP43 (Protein Data Bank [PDB] IDs: 5W3N, 6WQK, and 6N3C). And two structures from irreversible amyloid Tau and Orb2. Tau is associated with Alzheimer's pathogenesis, and the presented structure (PDB ID: 5O3T) was made by seeding purified protein with extracts from the brain of a deceased patient with Alzheimer's disease. Orb2 (PDB ID: 6VPS) from *Drosophila melanogaster* forms stable amyloid fibrils associated with memory formation in flies and was purified from fly brains. Qualitatively, the structures of proteins associated with dynamic MLOs (FUS, hnRNPA2, and TDP43) have more LARKS and have more kinks in their backbones when compared with the stable amyloids—disease associated (Tau) or functional amyloid (Orb2). LARKS, low-complexity amyloid-like reversible kinked segment; LCD, low-complexity domain.

**Figure 5:**
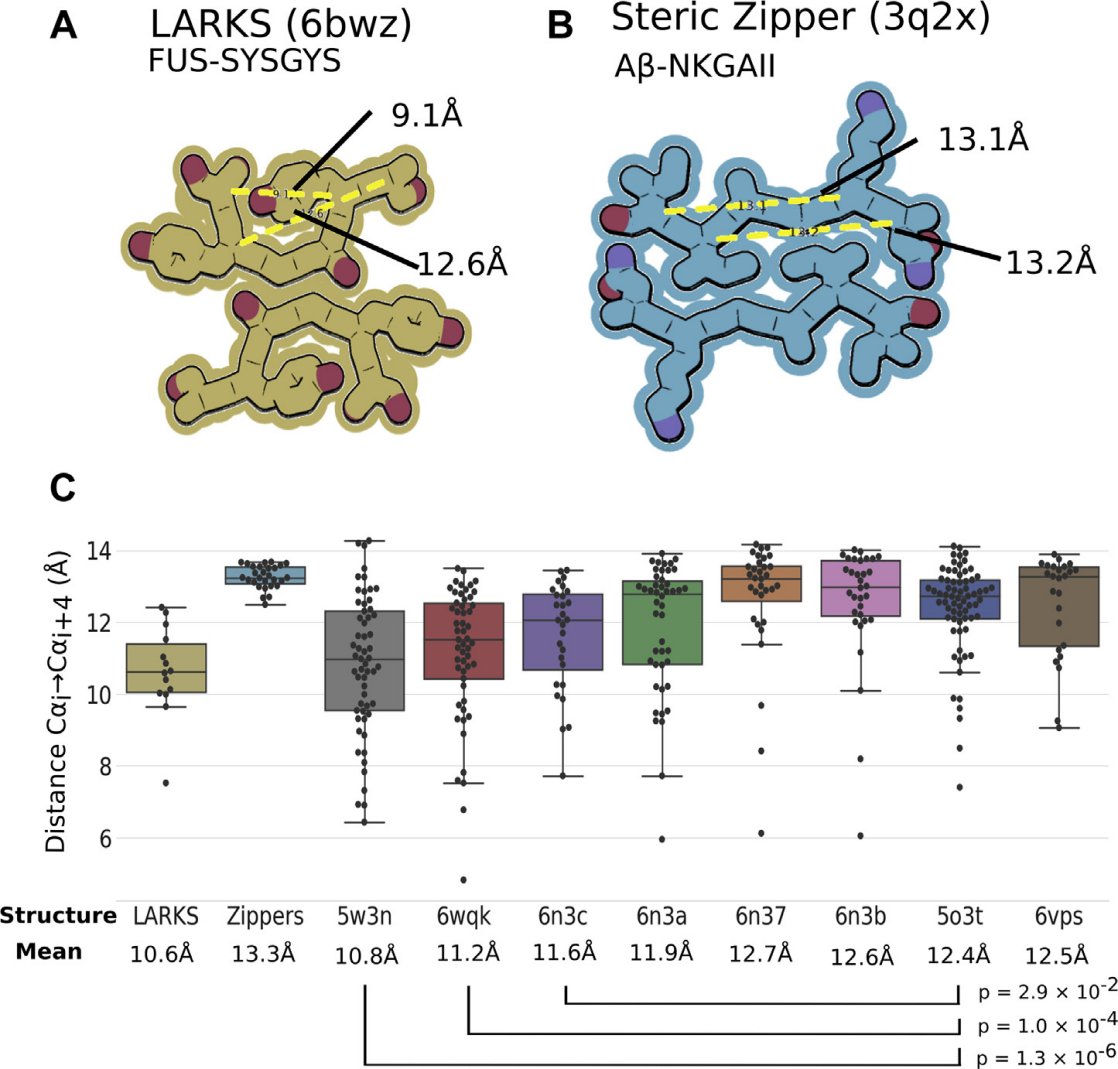
**LARKS in protein structure.** *A*, example of a LARKS structure, FUS-SYSGYS, shows the kinked backbone and minimal interface between mating sheets. Measuring all possible Cα_i_ → Cα_i + 4_ pairs finds distances of 12.6 and 9.1 Å, a result of the kinked backbone. *B*, an example of a steric zipper, Aβ-NKGAII, to show the extensive mating interface between the extended and pleated β-sheets. Cα_i_ → Cα_i + 4_ distances are 13.1 and 13.2 Å because the regular spacing from the β-sheet structure. *C*, *box* and whisker plots of Cα_i_ → Cα_i + 4_ distances. Each *black point* is a single distance measurement from either a sample of LARKS crystal structures, steric zipper structures, or the full-length proteins shown in Figure 4 as well as additional TDP43 structures in Fig. S2. The LARKS-rich LCD amyloids (FUS-5w3n, hnRNPA2-6wqk, and TDP43-6n3c) have significantly closer Cα_i_ → Cα_i + 4_ distances than found in irreversible amyloids (Tau-5o3t and Orb2-6vps) reflecting how LARKS affect protein structure by interrupting β-sheets. LARKS, low-complexity amyloid-like reversible kinked segment; LCD, low-complexity domain.

*S. cerevisiae* does not show enrichment for LARKS ∩ LCD proteins in the proteome (Fig. S1), but when GO analysis is applied to proteins with the most LARKS ∩ LCD residues, familiar GO terms are returned for mRNA binding, P bodies, and SGs (Fig. 3 and Table S2). This finding appears to contrast slightly with our findings that LARKS-rich proteins do not significantly overlap with LCD-containing proteins in *S. cerevisiae* (Fig. S1). We believe discrepancy arises because while there are not many LARKS ∩ LCD residues in *S. cerevisiae*, looking for proteins with LARKS ∩ LCD residues finds proteins with LCDs, and GO terms may highlight an overlap of function of LCDs even if they are not LARKS rich. If that is the case, then the *S. cerevisiae* proteome does not enrich for LARKS in LCDs, but GO indicates that LCD-containing proteins might serve similar functions for mediating phase separation as in *H. sapiens* but using different motifs than LARKS. Yeast SGs are less dynamic and more resemble aggregates than human SGs (25), which might stem from the LCDs in *S. cerevisiae* being more predisposed to forming steric zipper motifs over LARKS. We lack the overlapping datasets to do a direct comparison of steric zippers and LARKS in LCDs of *H. sapiens* and *S. cerevisiae*, but we performed a qualitative comparison of well-known proteins from each to illustrate relationship of LARKS and zippers in these organisms (Fig. S4 and Table S1).

We examined the LARKS and steric zipper content of human prion-like proteins with LCDs hnRNPA1, hnRNPA2, TDP43, and FUS to the LCDs of yeast prion proteins, Ure2 and Sup35. The LCDs of both the *H. sapiens* and the *S. cerevisiae* proteins have higher LARKS content than the protein as a whole (Fig. S4 and Table S1). To gauge the relative abundance of LARKS to zippers, we looked at the ratio of LARKS:zipper residues in the LCDs. In all *H. sapiens* prion-like proteins, the ratio of LARKS:zippers is above one, indicating a relative abundance if LARKS. All yeast prion proteins had LCDs with a LARKS:zipper ratio below one, implying a preference for zipper motifs. The human prion-like proteins are also notable in having far more LARKS than zippers when compared with other amyloid or globular proteins (Fig. S4), which reflects our finding that of the more dramatic enrichment of LARKS in human LCD proteins compared with yeast (Fig. S1). These data from a small and arbitrary sample illustrate how *S. cerevisiae* may use zippers to form more solid-like MLOs, but a larger comparison is needed. So, while LCDs seem common on proteins involved in MLOs, the LARKS content may tune material properties of condensates.

The proteome and organism in our analysis that most resembles *S. cerevisiae* is *P. falciparum*, which is a single-celled eukaryote with extensive LCDs that are poor in glycine and rich in asparagine residues (Fig. 2). GO analysis for LARKS-rich proteins yielded a term for catalytic activity, but we believe this may be spurious and reflects a few LCDs with a low number of LARKS within them because *P. falciparum* has far fewer proteins with LARKS ∩ LCD residues (Fig. 3). The proteome of *M. tuberculosis* has a population of proteins that are uniquely replete with LARKS ∩ LCD residues and returns unclassified GO terms. These proteins belong to the PE_PGRS family, a mysterious family of proteins with Gly–Ala-rich LCDs that decorate the outside of the cell wall (26). In fact, Gly–Ala repeat inclusions are associated with C9orf72-related amyotrophic lateral sclerosis and sequester cell machinery (27, 28). We find it striking that the LARKS ∩ LCD proteins on an obligate intracellular parasite are exposed to the host cell's cytoplasm. It is enticing to speculate that these LARKS-rich proteins may have coevolved with *H. sapiens* in order to interact with LARKS-rich protein host cells to help virulence. *P. falciparum* is also an obligate intracellular parasite, but compared with *M. tuberculosis*, the proteome is largely devoid of LARKS in LCDs. In the *P. falciparum* life cycle, the parasite is endocytosed by a red blood cell. Once in the vacuole, the parasite modifies the environment within to better suit it but is not exposed to the intracellular environment. This contrasts with the *M. tuberculosis* parasite, which can escape phagosomes to interact with the host cytoplasm (29). LARKS-rich proteins in humans are largely intracellular, involved in MLOs, and the LARKS-rich proteins displayed on the *mycobacterium* cell wall would be available to interact with the host machinery. Both parasites have extensive LCDs that must aide in virulence, but as their location and LARKS content is different, their mechanism of actions is likely different as well.

Taken together, the variable LARKS content of LCDs may reflect the utility and physical behavior of that protein. For example, the LARKS-rich LCDs of human SG proteins seem to be more liquid like than the LARKS-poor yeast SG proteins (25), which behave more like solid aggregates and are more prone to form steric zippers (Fig. S4 and Table S1). We reasoned that this difference in behavior could be reflected in structure since LARKS interrupt regular β-sheets, and to this end, we made use of the recent surge of amyloid structures determined by cryo-EM and solid-state NMR to interpret the effect that LARKS have on fibril structure. We opted to qualitatively compare the structures of putative functional amyloids of SG-associated proteins FUS and hnRNPA2 that have numerous LARKS—11 and 9, respectively—compared with the structure of Tau from disease-related amyloid that only has two LARKS (Fig. 4). The Tau structure is a continuous β-sheet that folds onto itself to create an extended steric zipper that is the backbone of irreversible fibrils found in Alzheimer's disease. The FUS and hnRNPA2 structures have a kinked backbone that interrupts the formation of any extended β-sheet, reflecting the number of LARKS in those structures.

How “kinked” a structure is can be roughly quantified by measuring the distance between Cα_i_ and Cα_i + 4_. The pleated β-sheets in steric zippers have a consistent distance around 13.3 Å, compared with LARKS where the kinks can reduce this distance to 7.5 to 12.6 Å (Fig. 5). The average Cα_i_ → Cα_i + 4_ distances in FUS and hnRNPA2 structures are significantly shorter (10.8 and 11.2 Å, respectively) than in Tau (12.4 Å), consistent with their respective LARKS content (Figs. 4 and Figure 1, Figure 5). The limited interfaces created by LARKS are associated with less stable amyloid-like fibrils (15), fitting given the reversibility of FUS and hnRNPA2 fibrils (4, 12, 30). This structural comparison of putatively functional and reversible amyloid fibrils hnRNPA2 and FUS (3) to Tau supports that LARKS alter structure in a way that is compatible with the biology of the different amyloid fibrils.

Not all functional amyloids are readily reversible, as is the case with Ordb2 from *D. melanogaster*. Orb2 forms a stable amyloid to aid in memory formation—stable amyloid is important to retain the memory over time (31). The fibril-forming sequence of Orb2 contains an LCD that overrepresents glutamine residues but has no predicted LARKS (Figs. 4 and 5). The cryo-EM structure of Orb2 amyloid fibril revealed a nearly perfect steric zipper of interdigitated glutamine residues. While a functional amyloid, Orb2 is better served by being irreversible, and this behavior is reflected in the lack of LARKS in the Orb2 sequence.

This correlation of LARKS with kinked structures even holds within the same LCD. There are now two structures of extended segments of the FUS LCD and four of the TDP43 LCD (32, 33, 34). The segments with more LARKS—Protein Data Bank (PDB) IDs: 5W3N and 6N3C—are more kinked than ones with fewer as judged by shorter Cα_i_ → Cα_i + 4_ distances (Figs. S2 and S3). FUS and other SG proteins form hydrogels that can be reversed several times before becoming irreversible (12, 30). It may be that the more kinked fibrils formed by LARKS may represent the labile form that may be a kinetic trap that ultimately yields to the more thermodynamically stable irreversible fibril core over time.

In summary, our findings support the hypothesis of LARKS as structural motifs in LCDs that can help mediate phase transitions and MLO organization in biology. We find LARKS abundant in species that have dynamic MLOs (*e.g.*, *D. melanogaster*, *H. sapiens*), sparse in species that have less dynamic SGs (*S. cerevisiae*), and suspiciously abundant in intracellular parasite proteins that are exposed to the cytoplasm (*M. tuberculosis*). LARKS are not universal in all LCDs (*e.g.*, *S. cerevisiae*, *P. falciparum*) and not in all proteins that undergo phase transitions. LARKS are one strategy that organisms have adopted for organizing MLOs, but not all proteins that phase separate need LARKS. Instead LARKS may tune the material properties of the condensates they are in by providing weak interactions for phase separation while avoiding stable aggregation mediated by steric zippers. The degree to which β-sheet structures form in MLOs remains debated (35), but we show that amyloid-like structures rich in LARKS have kinked structures, and the kinked structures follow the principle of protein negative design by interrupting exposed β-edges, thereby preventing pathogenic aggregation in MLOs (36). LARKS appear to be a functional structural motif coopted through evolution by widely divergent species.

All the data presented in this work are available at LARKSdb. This permits researchers to submit their own proteins for LARKS predictions.

## Experimental procedures

### Proteome origin

All proteomes used were UniProt reference proteomes. The following proteomes and date of downloads were used: *E. coli*: September 4, 2017; *M. tuberculosis*: April 4, 2016; *S. cerevisiae*: April 4, 2016, *P. falciparum*: April 25, 2016; *D. melanogaster*: April 4, 2016; and *H. sapiens*: March 28, 2016. Any protein sequence with a letter not in the 20 natural amino acids was discarded before analysis.

### Identifying LARKS

LARKS were identified using the methods outlined by Hughes *et al.*, 2018. The computational methods identify six-residue segments that are predicted to form LARKS segments. Briefly, the sequence of a new protein is broken into six residue segments (five residues overlap with an adjacent segment). The side chains for the sequence of the segment are computationally grafted onto a threading backbone of a known LARKS structure, and energy minimization using a Rosetta energy score is carried out. This is repeated for three separate LARKS structures (FUS-SYSGYS, FUS-STGGYG, and hnRNPA1-GYNGFG; PDB IDs: 6BWZ, 6BZP, and 6BXX). If the resulting segments score is below a threshold considered favorable for any of the three backbones; then the segment is considered to be a LARKS segment. When counting the number of residues found in LARKS, we consider any residue within the six-residue LARKS segment as a LARKS residue.

### Identifying LCDs

Residues in LCDs were identified by using the SEG algorithm (5). If 25 residues in a row were low in complexity as predicted by SEG, it was considered to be an LCD within a protein. Regions of low-complexity shorter than 25 residues were not considered to be in LCDs in our analysis.

### Identifying IDRs

IDRs were identified using GlobPlot 2.0 software (GlobPlot/GlobPipe) (23). The given script was downloaded and used to search proteomes for predicted globular regions. Any residue predicted to be in a globular region using the recommended settings was considered to be globular, and all other residues predicted to be in IDRs in our analysis.

### Computational overlap of predicted residues

All residues were given binary scores (either belonging to a category or not) in each category: LARKS, LCDs, and IDRs. Then residues in overlapping categories could be identified: LARKS in LCDs, LARKS in Glob, and LCD in Glob. Enrichment of overlapping classes of residues (*e.g.*, LARKS in LCDs) was calculated by finding the actual number of residues considered to be both LARKS and in LCDs within a proteome and comparing this to an expected number.

The “actual overlap” was found by dividing the number of overlapping residues in a category (*e.g.*, LARKS ∩ LCD) by the number of residues in the proteome. We then used bootstrapping techniques to find a 95% confidence interval for the actual value to lie in. To find the 95% confidence interval for LARKS in LCDs, we did 100 rounds of bootstrapping where residues equal to the number of LCD residues were drawn, with replacement, and the number of LARKS ∩ LCD residues from this sample was counted to find the sample LARKS ∩ LCD value. From the resulting 100 rounds of bootstrapping, a distribution of the average of LARKS ∩ LCD residues drawn from the LCD residue pool was made. The 2.5 and 97.5 percentiles were chosen to make the 95% confidence interval in Table 1. These 95% confidence intervals reflect the variance of the underlying data.

The expected overlap was found for each proteome by multiplying the probability of residues in one category (*e.g.*, LARKS) by the probability of a residue being in another category (*e.g.*, LCDs) to get the expected LARKS ∩ LCD. The law of large numbers dictates this is a reasonable null hypothesis because our sample sizes are in the millions of residues. If the expected value is below or above the 95% confidence interval for the actual value, then we can consider that protein to be enriched or depleted in LARKS in LCDs, respectively. This process is repeated to see for the following comparisons: LARKS in LCD regions, LARKS in Glob regions, and LCD in Glob regions.

### Bootstrapping methodology

For each proteome, we computed the average number of LARKS per 100 residues across the proteome. Any protein that has more than the average (given in the table below) for the organism was considered LARKS rich.

**Table:** 

Species	Average no. of LARKS per 100 residues
*E. coli*	2.2
*M. tuberculosis*	3.1
*P. falciparum*	0.9
*S. cerevisiae*	1.7
*D. melanogaster*	2.2
*H. sapiens*	2.2

The number of proteins with an LCD and considered LARKS rich was counted for each proteome to give the actual value (Fig. 1). Then, bootstrapping was done by randomly drawing proteins, with replacement, from the proteome equal to the actual number of proteins with LCDs that were counted from that proteome. From that sample, the number of LARKS ∩ LCD proteins was counted. This process was repeated 10,000 times to build a distribution of numbers of LARKS ∩ LCD proteins that are found in the random samples of the proteome equal in size to the number of LCD proteins. The actual number of LARKS ∩ LCD proteins was counted and plotted on the histogram to see if it was above, below, or within the distribution—actual values are given in the table below (Fig. S1). A *p* value was calculated by counting the number of random samples that had values that exceeded the actual number of LARKS ∩ LCD proteins and dividing by the number of samples (except for *P. falciparum* where the number of samples with fewer LARKS ∩ LCD proteins to find depletion of LARKS ∩ LCD in LCD proteins).

**Table:** 

Species	No. of samples > LARKS ∩ LCD actual	No. of samples < LARKS ∩ LCD actual
*E. coli*	3	9997
*M. tuberculosis*	0	10,000
*P. falciparum*	10,000	0
*S. cerevisiae*	2884	7116
*D. melanogaster*	0	10,000
*H. sapiens*	0	10,000

### GO

GO was done on the top 5% of LARKS-rich proteins in each proteome with LARKS-rich defined as the average number of LARKS per 100 residues of a protein. The list of UniProt IDs for these top 5% of proteins was submitted to the Panther GO server to see enriched terms (37). Relevant GO terms were selected arbitrarily for Figure 3, and the complete list of GO term hits is provided in Table S2.

## Data availability

All the threading data are available on the LARKSdb server (http://servicesn.mbi.ucla.edu/LARKSdb/).

## Supporting information

This article contains supporting information.

## Conflict of interest

D. S. E. is SAB chair and equity holder in ADRx, Inc. All other authors declare that they have no conflicts of interest with the contents of this article.
