# Supplementary material for: Prevalence and species distribution of the low-complexity, amyloid-like, reversible, kinked segment structural motif in amyloid-like fibrils
Source: J Biol Chem. 2021 Sep 16;297(4):101194. doi: 10.1016/j.jbc.2021.101194 (PMC8551513; doi:10.1016/j.jbc.2021.101194)
Supplement: Supplemental Figs. S1–S4 and Table S1 [file mmc1.docx]

**Supporting Information for**

**Prevalence and species distribution of the low-complexity, amyloid-like, reversible, kinked segment structural motif in amyloid-like fibrils**

**Authors:** **Michael P. Hughes*, Lukasz Goldschmidt#, David S. Eisenberg****


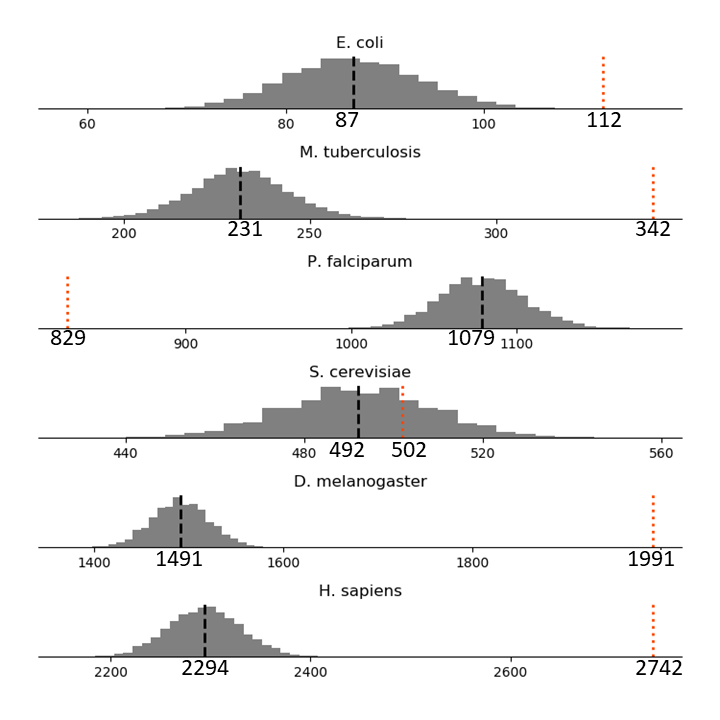


**Supplemental Figure 1.** Bootstrapping statistics show LARKS-rich and LCD-containing proteins significantly overlap in some organisms. For each proteome shown in Figure 1, the number of LARKS∩LCD proteins are shown by the red line. Then we drew proteins randomly with replacement from the species proteome equal to the number of proteins with LCDs in the proteome. From that random sample the number of LARKS∩LCD proteins that had an LCD and were LARKS-rich were counted, and this process was repeated 10,000 times to create a distribution of averages. The distribution average is given by the black line and the actual average of LARKS∩LCD below it. The data indicate that LARKS are significantly enriched in LCD proteins for all species except *P. falciparum* and *S. cerevisiae*. In *S. cerevisiae* the actual number of LARKS∩LCD proteins is well within the expected distribution generated from bootstrapping, and in *P. falciparum* proteins considered LARKS-rich are less likely to have and LCD than expected by random chance.


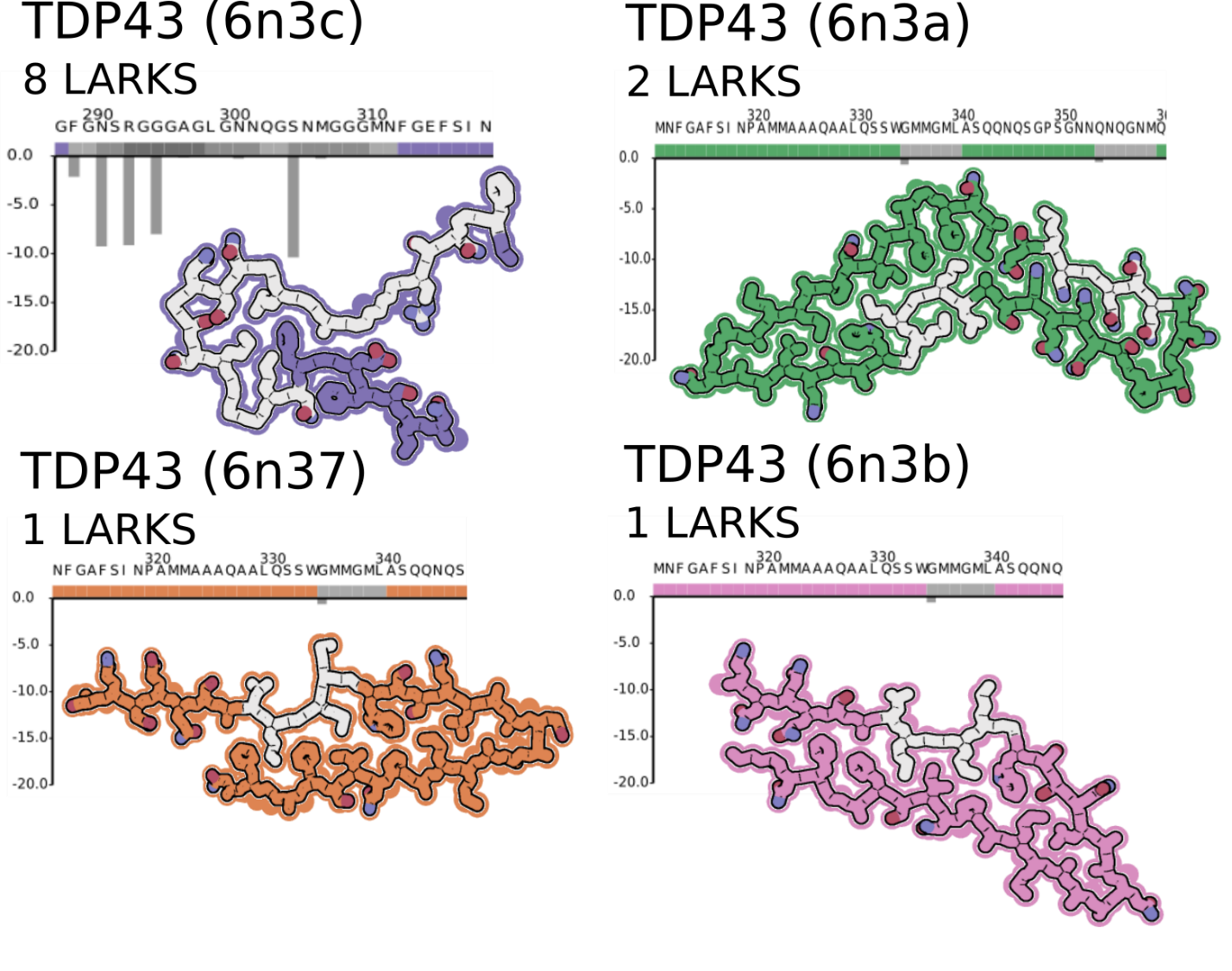


**Supplemental Figure 2.** Three additional structures of polymorphic amyloid fibrils TDP43 from different regions of the LCD (PDBIDs: 6n37, 6n3a, and 6n3b) with few LARKS compared to a LARKS-rich region of the LCD (6n3c) to highlight the kinked backbone created by LARKS in 6n3c.


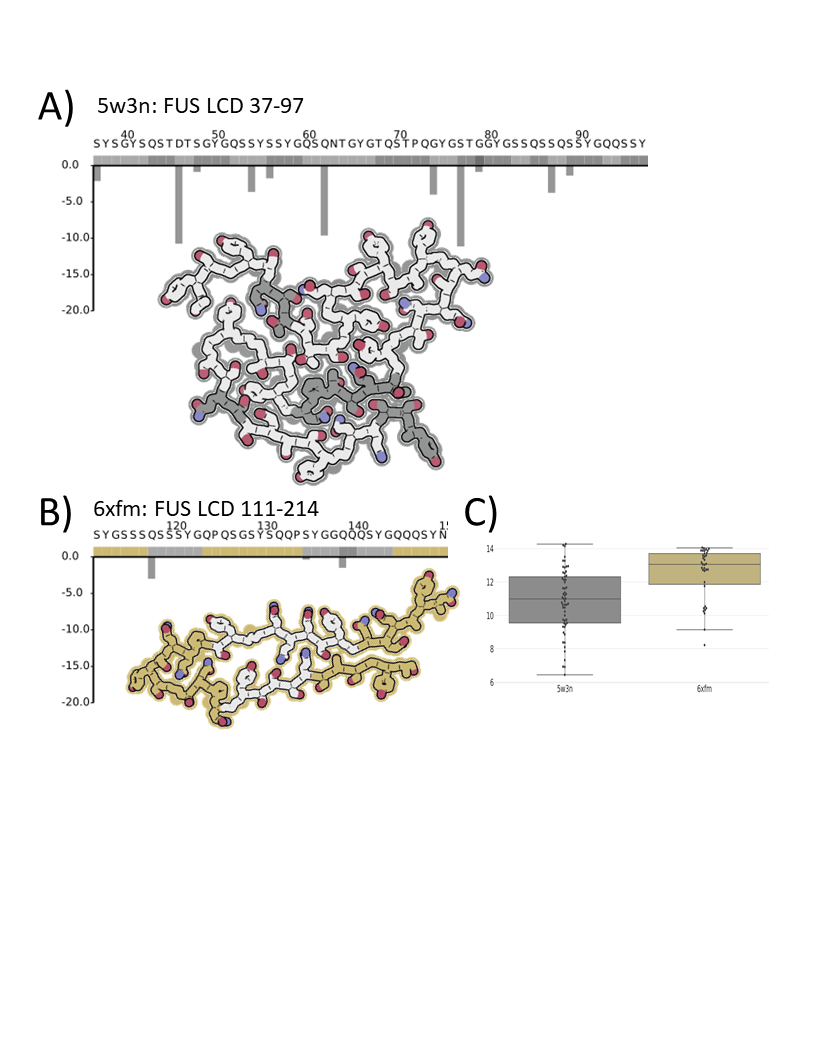
**Supplemental Figure 3**. FUS LCD amyloid fibril structure comparisons. A) The FUS structure 5w3n, determined by ssNMR, consists of LCD residues 37-97. This segment contains 11 LARKS and a dramatically kinked backbone. B) The FUS structure 6xfm, determined by cryoEM, consists of residues 111-150, but only has 3 LARKS. C) Box plot of Cα_i_ → Cα_i+4_ distances from FUS structures shows that the average distance is shorter for the LARKS-rich 5w3n structure compared to the LARKS-poor 6xfm structure reflecting the effect of LARKS on the amyloid backbone.


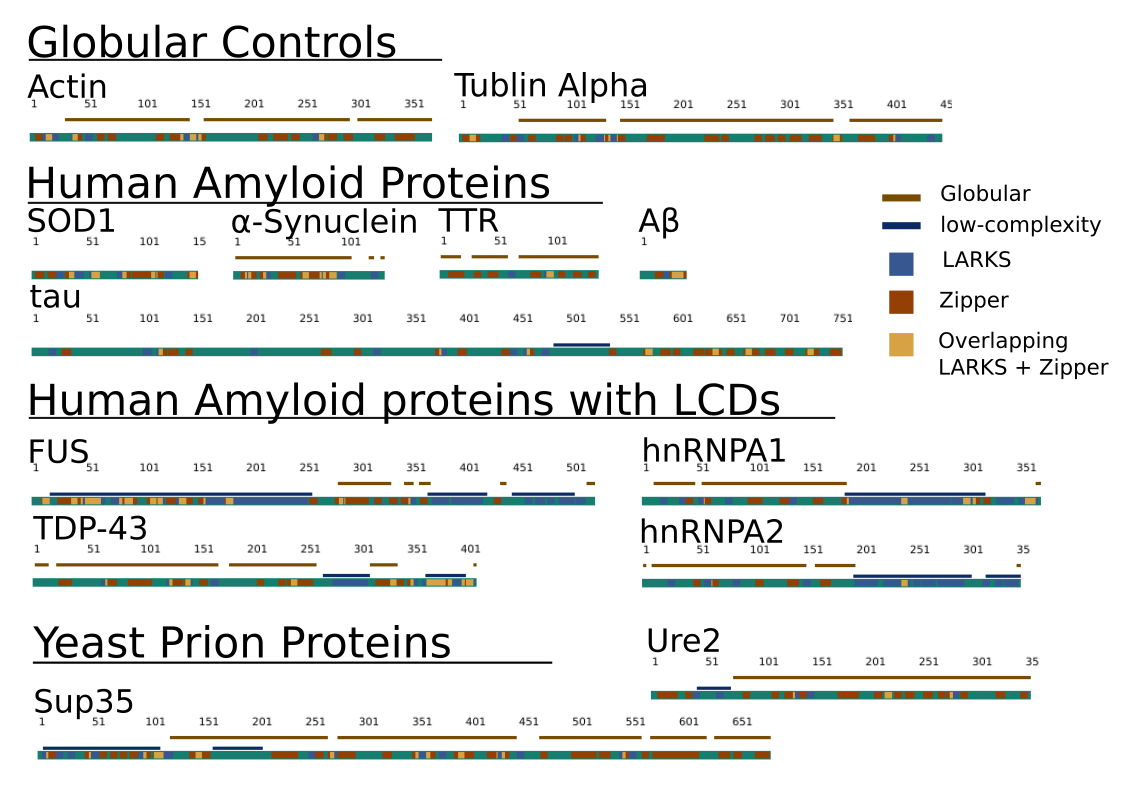


**Supplemental Figure 4**. **Human prion-like proteins have LCDs uniquely rich in LARKS.** Human prion-like proteins have more LARKS in their LCDs than other proteins in a qualitative comparison. Proteins are depicted as bars, scaled to the length of the protein. The bands of color are blue for residues in LARKS, red for residues in Zippers, and green for residues in both. Regions that are predicted to be globular or and LCD are highlighted by red and blue lines respectively that are above the protein schematic, but below the sequence numbering. A human actin and tubulin sequence were chosen as examples of normal, globular proteins. Human amyloid proteins SOD1, α-synuclein, transthyretin (TTR), Aβ, and tau are depicted as amyloid proteins that do not have LCD regions and are low in LARKS. FUS, TDP43, hnRNPA1, and hnRNPA2 are all found as amyloid inclusions in some neurodegenerative diseases but are also considered membraneless organelle constituents. They all have LCDs with abundant LARKS. These contrast to the yeast prion proteins that have LCDs that are more abundant in steric zippers.

| Protein Name | # residues | #LARKS residues | #Zipper residues | Fraction LARKS residues | Fraction Zipper residues | Residue Ratio LARKS:zippers |
| --- | --- | --- | --- | --- | --- | --- |
| actin | 375 | 57 | 143 | 0.15 | 0.38 | 0.4 |
| tubulin alpha | 451 | 60 | 162 | 0.13 | 0.36 | 0.37 |
| Aβ | 42 | 17 | 22 | 0.4 | 0.52 | 0.77 |
| α-synuclein | 140 | 42 | 67 | 0.3 | 0.48 | 0.63 |
| SOD1 | 154 | 54 | 75 | 0.35 | 0.49 | 0.72 |
| transthyretin | 147 | 14 | 63 | 0.1 | 0.43 | 0.22 |
| Tau | 758 | 80 | 185 | 0.11 | 0.24 | 0.43 |
| hnrnpa1 | 372 | 180 | 85 | 0.48 | 0.23 | 2.12 |
| hnrnpa1 LCD | 187 | 150 | 42 | 0.8 | 0.22 | 3.57 |
| hnrnpa2 | 353 | 138 | 44 | 0.39 | 0.12 | 3.14 |
| hnrnpa2 LCD | 161 | 112 | 6 | 0.7 | 0.04 | 18.67 |
| fus | 526 | 268 | 182 | 0.51 | 0.35 | 1.47 |
| fus LCD | 214 | 128 | 111 | 0.6 | 0.52 | 1.15 |
| tdp43 | 414 | 120 | 171 | 0.29 | 0.41 | 0.7 |
| tdp43 LCD | 152 | 90 | 67 | 0.59 | 0.44 | 1.34 |
| sup35 | 685 | 120 | 306 | 0.18 | 0.45 | 0.39 |
| sup35 LCD | 135 | 51 | 56 | 0.38 | 0.41 | 0.91 |
| ure2p | 354 | 38 | 137 | 0.11 | 0.39 | 0.28 |
| ure2p LCD | 89 | 12 | 27 | 0.13 | 0.3 | 0.44 |

Globular control

proteins

Amyloid

proteins

Human prion-like proteins

Yeast Prions proteins

**Supplemental Table 1.** Summation of a qualitative comparison of LARKS and steric zipper residues in proteins of interest shows that human prion-like proteins (hnRNPA1, hnRNPA2, FUS, and TDP43) have a higher ratio of LARKS to zippers compared to Globular control proteins (e.g. actin and tubulin alpha), amyloid proteins (e.g. Aβ, α-synuclein, SOD1, transthyretin, and Tau), and yeast prion proteins (e.g. sup35 and ure2p). The LCDs of human prion-like proteins have a higher ratio of LARKS:zipper residues compared to the whole protein. Yeast prion protein LCDs have more zipper residues than LARKS residues while human prion-like protein LCDs have more LARKS than zipper residues. Data for the LCDs of prion and prion-like proteins highlighted in gray.
